# Factors influencing registered nurses’ attitudes toward and literacy in artificial intelligence: a cross-sectional systematic review

**DOI:** 10.3389/fpubh.2026.1997701

**Published:** 2026-10-23

**Authors:** Yingqi Dai, Xili Zhao, Xiao Pan, Jiaxin Wei

**Affiliations:** 1 The Second Affiliated Hospital of Chongqing Medical University, Chongqing, China; 2 Acute and Critical Care Nursing Innovation and Translation Engineering Research Center of Chongqing Education Commission of China, Chongqing, China; 3 Chongqing Geriatric Specialized Nursing Clinical Diagnosis and Treatment Center, Chongqing, China

**Keywords:** artificial intelligence, attitude, influencing factors, literacy, nurses

## Abstract

**Background:**

Presently, artificial intelligence technology is being extensively applied across clinical nursing practice. However, whether this technology can be deeply integrated into clinical workflows depends largely on nurses’ attitudes and literacy regarding artificial intelligence. Consequently, conducting an in depth exploration of core factors shaping registered nurses’ artificial intelligence attitudes and literacy holds significant importance for driving the rapid development of intelligent nursing.

**Methods:**

This study strictly adhered to PRISMA guidelines. Systematic searches were conducted across seven English databases, namely PubMed, Web of Science, Medline, Embase, The Cochrane Library, IEEE Xplore, and CINAHL, alongside three Chinese databases, including China National Knowledge Infrastructure (CNKI), Wanfang Data, and VIP Database. The search timeframe ranged from database inception through May 30, 2026. Following screening according to inclusion and exclusion criteria, independent factors influencing attitudes and literacy were extracted. Employing a narrative synthesis approach, these influencing factors were categorized, summarized, and narratively integrated across distinct thematic domains.

**Results:**

A total of 30 studies involving 12,958 registered nurses were included in this systematic review. The majority of the studies were conducted in China and Turkey. The independent factors influencing registered nurses’ attitudes toward artificial intelligence included educational attainment, age, length of service, gender, professional title/nursing level, hospital tier/category, AI awareness/understanding/knowledge, AI training experience, AI usage experience, artificial intelligence anxiety, and digital leadership. The factors independently affecting nurses’ artificial intelligence literacy included leadership style, AI training experience, and usage experience/frequency.

**Conclusion:**

Current evidence indicates that key modifiable factors to improve nurses’ attitudes toward and literacy in artificial intelligence include AI-related training, practical hands-on experience, and organizational leadership, while technical anxiety should not be overlooked. Compared with demographic characteristics, educational and experiential interventions appear more effective in enhancing nurses’ artificial intelligence literacy levels. Nursing managers should actively implement smart training programs, increase opportunities for nurses to apply new technologies in clinical practice, and formulate relevant policies regarding data privacy protection and accountability division, thereby promoting the safe and effective integration of AI technology into patient care.

**Systematic review registration:**

https://www.crd.york.ac.uk/PROSPERO/recorddashboard

## 1 Introduction

With the rapid development of information technology, artificial intelligence (AI) technology has become a critical driving force in transforming modern healthcare. AI technology is fundamentally changing the delivery of medical services, spanning disease diagnosis, treatment selection, and patient monitoring, thereby significantly enhancing the accuracy and efficiency of healthcare delivery (1). By solving complex clinical problems and optimizing resource allocation, AI plays a vital role in improving patient outcomes and enhancing overall healthcare quality (2). As technological integration deepens, healthcare providers must possess the appropriate capabilities to better adopt these technologies in clinical practice (3). As frontline clinical personnel, nurses constitute the largest professional group in the healthcare system, and the deep integration of AI technology with clinical practice presents them with unprecedented opportunities (4). AI algorithms are widely applied in fall prevention, pressure injury prevention, and early identification of sepsis (5, 6). AI-driven clinical decision support systems (CDSS) provide nurses with evidence-based recommendations by analyzing patient data (4); Natural language processing (NLP) systems alleviate administrative burdens through automated clinical documentation (3, 7), while the introduction of nursing robots improves work efficiency, enabling nurses to focus more on patient-centered care (8).

Despite the broad application prospects of AI technology, the successful implementation of these technologies largely depends on nurses’ mastery of the technology and their acceptance of its application (9–12). Without a deep understanding of the perspective of frontline nurses, technological resources may be wasted, and potential ethical conflicts alongside a loss of humanistic nursing care may arise (13). Current research indicates that nurses generally hold positive and optimistic attitudes toward AI technology (9, 14). Most nurses recognize the immense potential of AI in improving diagnostic accuracy, optimizing personalized care, and reducing workload (14, 15). However, they also express strong concerns regarding data privacy and security, algorithmic bias, and the potential for technology to diminish interpersonal interactions and the essence of “humanistic care” (10, 13). In addition, nurses’ level of artificial intelligence literacy needs to be improved, as most nurses have limited understanding of the specific use or functions of artificial intelligence technology (3, 11, 13, 16, 17).

Existing research demonstrates that nurses’ attitudes toward AI and their level of artificial intelligence literacy are collectively influenced by various complex factors. Gender, age, and technical usage experience are significantly associated with AI literacy levels, with younger or tech-experienced nurses often displaying higher acceptance (12, 16). Meanwhile, the lack of systematic training support, high implementation costs, and distrust in technical reliability constrain nurses’ positive adoption of AI technology to varying degrees (11, 14). Current studies in this field remain fragmented, with disparate findings across different countries and regions. This study aims to systematically integrate existing evidence to investigate the factors influencing registered nurses’ AI attitudes and literacy, thoroughly discuss factors with independent significance, and explore differences among populations with distinct characteristics, thereby providing empirical evidence for the better integration of AI technology into clinical practice.

## 2 Methods

### 2.1 Design

This systematic review was conducted in accordance with the Preferred Reporting Items for Systematic Reviews and Meta-Analyses (PRISMA) guidelines (18). The protocol for this study was registered in PROSPERO under the registration number CRD420251207546.

### 2.2 Search methods

This systematic review involved a comprehensive search across seven English databases (PubMed, Web of Science, Medline, Embase, The Cochrane Library, IEEE Xplore, and CINAHL) and three Chinese databases (China National Knowledge Infrastructure [CNKI], Wanfang Data, and VIP). The search period extended from database inception to May 30, 2026. The search strategy combined subject headings (Subject Headings/MeSH) and free text terms using Boolean operators. Primary search terms encompassed “nurses,” “registered nurses,” “artificial intelligence,” “deep learning,” “attitudes,” “perceptions,” and “literacy.” Additionally, to ensure literature recall, a snowballing approach was used to manually trace the reference lists of included studies. Detailed search strategies for each database are available in Supplementary Table A.

### 2.3 Inclusion and exclusion criteria

Based on the PECOs framework (P: Population; E: Exposure; C: Comparison; O: Outcome; S: Study Design), the inclusion criteria were established as follows:

① Population: registered nurses;

② Exposure: utilization of artificial intelligence technology in clinical work environments;

③ Outcome measures: artificial intelligence attitudes or literacy evaluated as dependent variables, with reporting of at least one correlation between AI attitudes or literacy and other variables;

④ Study design: cross-sectional studies;

⑤ Language: English or Chinese.

The exclusion criteria were:

① Study design consisting of mixed methods, reviews, meta-analyses, commentaries, editorials, protocols, or conference abstracts;

② Study population involving mixed populations;

③ Full text unavailable even after contacting the corresponding authors.

### 2.4 Data abstraction

Literature search was conducted independently by two researchers based on the established search strategy. All retrieved records were imported into NoteExpress reference management software, and duplicate entries were eliminated using its deduplication feature. During the primary screening phase, two researchers independently screened titles and abstracts to exclude studies that clearly did not meet the criteria. Studies passing primary screening progressed to full text review, where they were rigorously evaluated against the predefined inclusion and exclusion criteria. Any disagreements during screening were resolved through discussion with a third researcher. Data extraction was performed independently by two researchers using a standardized Microsoft Excel template. Extracted variables included first author, publication year, country or region, sample size, mean age, female sample size and percentage, clinical department, measurement instruments, quality assessment, and key outcomes. Upon completion, extracted data were cross checked by both researchers, with any discrepancies arbitrated by a third researcher.

### 2.5 Synthesis

All included studies in this systematic review were cross-sectional designs. Owing to substantial heterogeneity in assessment instruments, sample characteristics, and variable definitions across the included literature, quantitative meta-analysis was deemed unsuitable. Consequently, narrative synthesis alongside the Synthesis Without Meta-analysis (SWiM) guidelines was employed to describe and synthesize the included studies (19, 20). First, a customized data extraction table was utilized to summarize and conduct descriptive analyses on the primary characteristics of the included literature. Second, we referenced the Unified Theory of Acceptance and Use of Technology (UTAUT) model proposed by Venkatesh et al. (21) to conceptually cluster the factors influencing registered nurses’ artificial intelligence attitudes and literacy. During the data extraction phase, a two-tiered evidence matrix categorizing “significant univariate analysis” and “significant multivariate regression analysis” was constructed to comprehensively display variable distribution and publication frequency. Finally, to ensure evidentiary strength and rigor, the reporting of results and the discussion section were grounded strictly in independent factors that retained statistical significance (*p* < 0.05) in regression analyses after controlling for confounding variables.

### 2.6 Quality appraisal

To ensure rigor and transparency in quality assessment, this study utilized the Joanna Briggs Institute (JBI) Critical Appraisal Checklist for Analytical Cross-Sectional Studies (22). This instrument comprises 8 appraisal items, each scored dichotomously as “Yes” (1 point) or “No” (0 points), yielding a total score ranging from 0 to 8 points. Quality appraisal was performed independently by two researchers, with any scoring discrepancies resolved through consultation and discussion with a third researcher (see Supplementary Table B).

## 3 Results

### 3.1 Search outcome

Our search across 10 databases including PubMed and Web of Science yielded 10,999 records. A total of 3,448 duplicate records were removed using automated reference management software features, and 17 records were excluded due to retraction or other reasons. The remaining 7,534 records underwent title and abstract screening independently by two researchers, leading to the exclusion of 7,213 irrelevant studies. Full text retrieval was sought for the remaining 321 records, of which 6 full texts were unavailable. The remaining 315 full text articles were rigorously evaluated against the eligibility criteria, resulting in the exclusion of 285 studies for the following reasons: absence of key outcomes (*n* = 260), mixed population (*n* = 9), non AI related topics (*n* = 5), letters (*n* = 5), commentaries (*n* = 3), and conference abstracts (*n* = 3). Ultimately, 30 studies were included in this systematic review (Figure 1).

**Figure 1:**
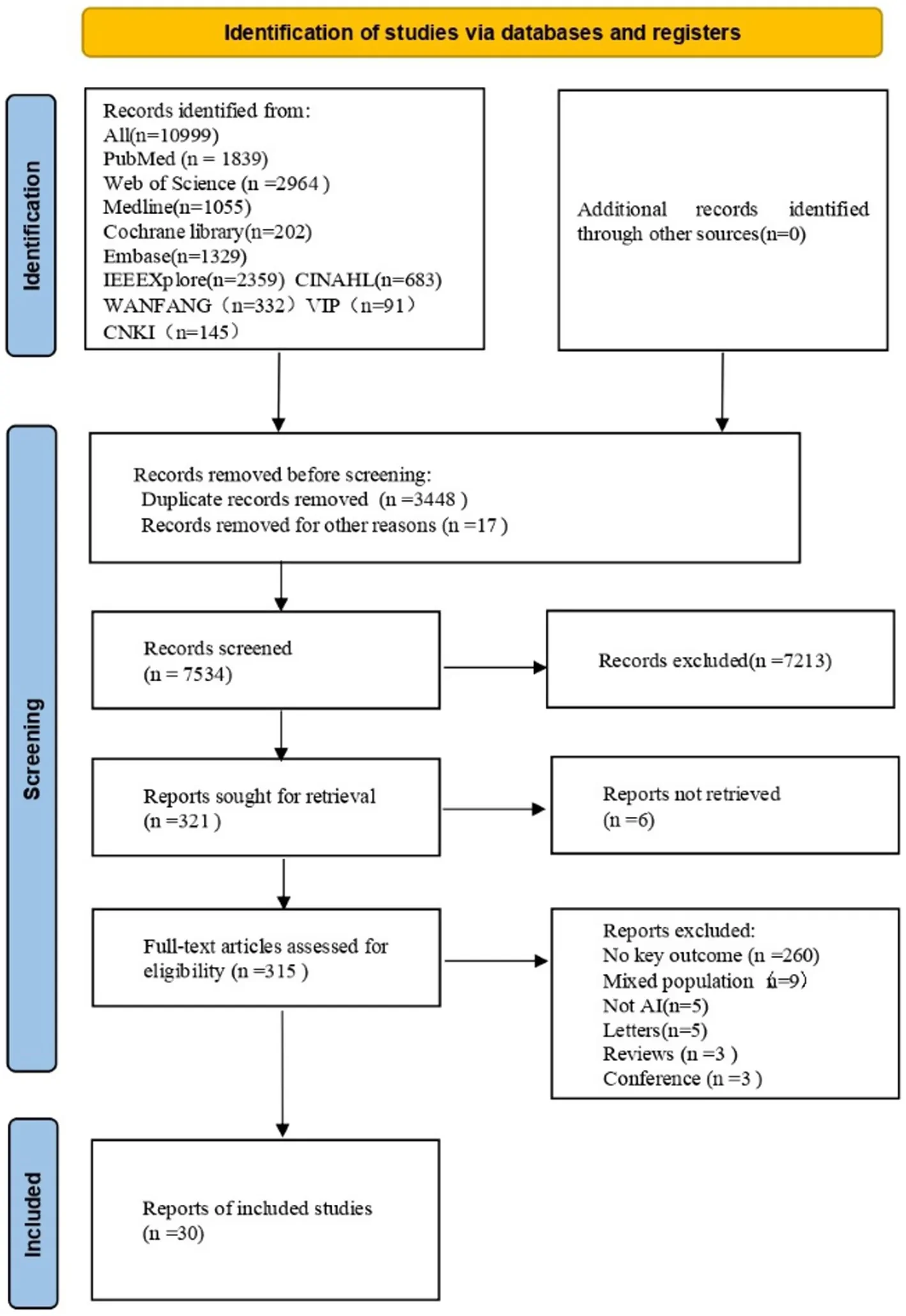
PRISMA flow diagram of the literature search and study selection process.

### 3.2 Characteristics of the studies

The 30 studies included in this review comprised a total of 12,958 registered nurses across multiple countries and regions, including China (*n* = 11), Türkiye (*n* = 12), Egypt (*n* = 2), Saudi Arabia (*n* = 2), Jordan (*n* = 2), and Iran (*n* = 1) (Figure 2). Two studies were published in Chinese and 28 in English. The scales measuring artificial intelligence attitudes were predominantly the General Attitudes Toward Artificial Intelligence Scale (GAAIS), along with the Attitude Scale for the Use of Artificial Intelligence Technologies in Nursing, TAME-ChatGPT, Artificial Intelligence Attitude Scale, AAAW, Nurses’ Attitudes Towards Artificial Intelligence Questionnaire, ASUAITIN-C, Attitude questionnaire, Artificial Intelligence Attitude Scale for Nurses, ASUAITIN, and other self-designed questionnaires. Scales assessing artificial intelligence literacy were mainly the Artificial Intelligence Literacy Scale (AILS), with only the study by Çoban et al. using the artificial intelligence literacy scale developed by Laupichler et al. (Table 1).

**Figure 2:**
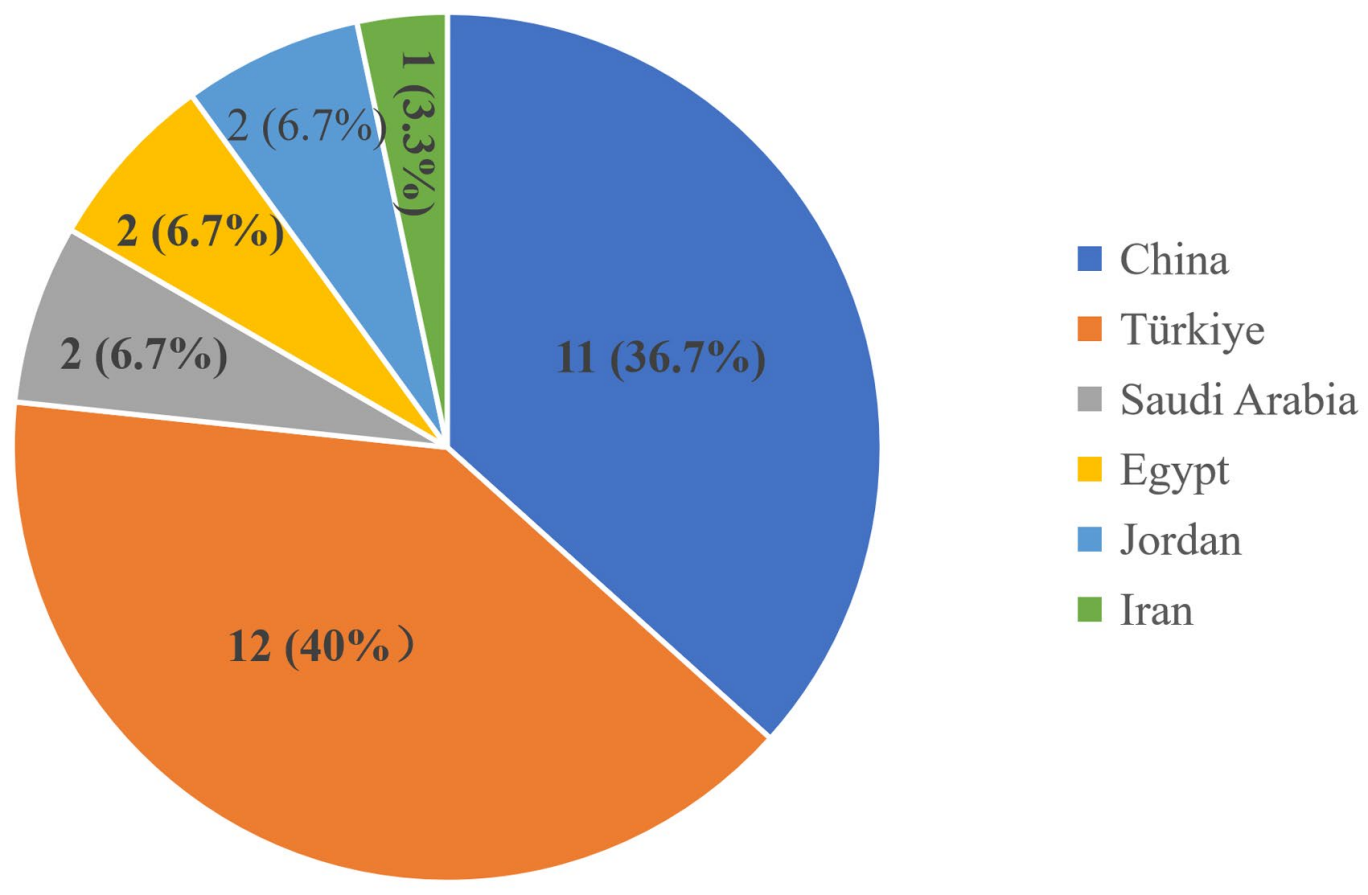
Geographic distribution of included studies by country (*n*, %).

**Table 1:** Baseline characteristics of included studies.

Author, year, Country	Sample (*n*)	Age (Mean ± SD)	Female (*n*, %)	Department	Measurement instruments	Quality (JBI)	Key outcomes
Deng et al., 2025, China (38)	280	NR	278 (99.3)	NR	Self-developed scale	High	AI attitude
Kong et al., 2025, China (40)	504	NR	432 (85.7)	NR	AILS	High	AI literacy
Liu et al., 2026, China (33)	221	NR	211 (95.5)	PICU	GAAIS; AILS	High	AI attitude; AI literacy
Taşçı et al., 2026, Türkiye (48)	368	NR	225 (61.1)	ICU, Operating room, Emergency, Surgical services	Attitude Scale for the Use of Artificial Intelligence Technologies in Nursing	Moderate	AI attitude
Kahraman et al., 2025, Türkiye (16)	505	31.55 ± 7.70	399 (70.0)	Operating room, Surgical clinic, Surgical intensive care, Surgical outpatient clinic, Emergency service	AILS	Moderate	AI literacy
Lin et al., 2025, China (49)	270	42.05 ± 9.84	261 (96.7)	NR	TAME-ChatGPT	High	AI attitude
Tarsuslu et al., 2025, Türkiye (25)	439	NR	331 (75.4)	NR	Artificial Intelligence Attitude Scale	High	AI attitude
Atalla et al., 2025, Egypt (50)	380	44.07 ± 6.93	319 (83.9)	Critical care units	GAAIS	High	AI attitude
Erkayıran et al., 2025, Türkiye (23)	388	33.82 ± 7.20	259 (66.8)	Internal services, Intensive care unit, Surgical services, Emergency service	GAAIS	Moderate	AI attitude
Alruwaili et al., 2024, Saudi Arabia (51)	220	NR	162 (73.6)	NR	GAAIS	Moderate	AI attitude
Li et al., 2026, China (32)	595	NR	464 (78.0)	NR	AAAW	High	AI attitude
Sayed et al., 2026, Jordan (52)	91	33.5 ± 6.3	66 (72.5)	Critical care units	Nurses’ Attitudes Towards Artificial Intelligence Questionnaire	Moderate	AI attitude
Xu et al., 2026, China (34)	436	NR	386 (88.5)	NR	ASUAITIN-C; AILS	High	AI attitude; AI literacy
Alrashedi et al., 2026, Saudi Arabia (26)	203	NR	74 (36.5)	Critical care units	GAAIS	High	AI attitude
Tuncer et al., 2024, Türkiye (53)	306	29.4 ± 6.6	260 (90.3)	Emergency room; Inpatient Service, Intensive care unit, Outpatient clinic, Dialysis Unit etc	GAAIS	Moderate	AI attitude
Zeng et al., 2025, China (54)	878	NR	826 (94.1)	Pediatrics, Obstetrics and gynecology, Non-inpatient wards, Adult general	GAAIS; AILS	High	AI attitude; AI literacy
Namdar et al., 2025, Iran (55)	389	32.81 ± 7.67	248 (63.8)	NR	Attitude questionnaire	Moderate	AI attitude
Cin et al., 2026, Türkiye (56)	267	27.36 ± 6.80	171 (64.0)	Internal Medicine Clinics, Surgery Clinics, Pediatric Clinic, Management	GAAIS	Moderate	AI attitude
Gürdap et al., 2026, Türkiye (57)	323	32.46 ± 7.32	138 (42.7)	intensive care units, internal medicine, surgical, emergency service	Artificial Intelligence Attitude Scale for Nurses	High	AI attitude
Liu et al., 2025, China (58)	450	NR	437 (97.1)	NR	AILS; GAAIS	High	AI attitude; AI literacy
Hacıalioğlu et al., 2025, Türkiye (59)	377	33.3 ± 7.17	341 (90.5)	NR	GAAIS	High	AI attitude
Özçevik Subaşi et al., 2025, Türkiye (43)	170	33.32 ± 7.17	154 (90.6)	Paediatric clinics	GAAIS; AILS	High	AI attitude; AI literacy
Alnazly et al., 2026, Jordan (30)	312	NR	168 (53.8)	NR	GAAIS	High	AI attitude
Çoban and Beydağ. 2025, Türkiye (60)	339	34.35 ± 9.37	290 (85.5)	operating rooms, surgical clinics, surgical intensive care units, wound and burn care units	Artificial Intelligence Literacy Scale	Moderate	AI literacy
Chen et al., 2025, China (27)	564	NR	526 (93.3)	internal medicine, surgery, emergency, intensive care unit, others	ASUAITIN	High	AI attitude
Atalla et al., 2024, Egypt (61)	415	44.04 ± 7.03	347 (83.6)	NR	GAAIS	High	AI attitude
Uludaşdemir et al., 2026, Türkiye (62)	246	28.45 ± 4.48	182 (74.0)	Children’s hospital within a city hospital	AILS	High	AI literacy
Nirgiz et al., 2026, Türkiye (63)	562	29.03 ± 7.73	454 (80.8)	NR	GAAIS	High	AI attitude
Tong et al., 2026, China (42)	1,644	NR	1,534 (93.3)	Medical ward, Surgical ward, Obstetrics and gynecology department, Pediatrics, Intensive care unit, Emergency department, Other units	AILS	High	AI literacy
Yu et al., 2025, China (64)	816	31.4 ± 6.7	788 (96.6)	NR	Self-developed scale	High	AI attitude

GAAIS, The General Attitudes Toward Artificial Intelligence Scale; AILS, Artificial Intelligence Literacy Scale; TAME-ChatGPT, Technology Acceptance Model Edited to Assess ChatGPT Adoption; AAAW, Attitudes towards AI Application at Work scale; ASUAITIN-C, The Chinese Version of the Attitude Scale Toward the Use of AI Technologies in Nursing; ASUAITIN, Attitude toward the usage of AI; NR, not reported.

### 3.3 Factors influencing registered nurses’ artificial intelligence attitudes and literacy

#### 3.3.1 Demographic factors

Demographic factors influencing registered nurses’ artificial intelligence attitudes included educational level (*n* = 15), age (*n* = 13), years of work experience (*n* = 11), gender (*n* = 9), professional title or nursing care level (*n* = 5), and marital status (*n* = 5). Factors confirmed by multivariate regression analysis to have independent effects included educational level (*n* = 4), age (*n* = 5), years of work experience (*n* = 2), gender (*n* = 3), and professional title or nursing care level (*n* = 3) (see Table 2). Marital status showed no independent effect. Sociodemographic factors associated with registered nurses’ artificial intelligence literacy included educational level (*n* = 4), years of work experience (*n* = 1), age (*n* = 2), gender (*n* = 2), and marital status (*n* = 2), none of which displayed independent influence in the multivariate analysis (see Table 3).

**Table 2:** Factors influencing artificial intelligence attitudes.

Category of factors	Factors	Univariate analysis (*n*, references)	Multivariate analysis (*n*, references)
Demographic factors	Educational level	15 (23, 26, 27, 34, 38, 49–51, 53, 54, 56–58, 63)	4 (26, 27, 38)
	Age	13 (25–27, 30, 34, 38, 43, 48, 50, 51, 53, 58)	5 (25–27, 30, 34)
	Years of work experience	11 (30, 27, 61, 50, 56, 23 57, 26, 63, 25, 48)	2 (25, 26)
	Gender	9 (23, 26, 27, 30, 43, 48, 49, 51, 57)	3 (26, 27, 30)
	Professional title or nursing care level	5 (27, 34, 38, 58)	3 (27, 34, 58)
	Marital status	5 (26, 27, 49, 57, 63)	0
Organizational and occupational characteristics	Hospital grade or type	8 (23, 27, 33, 34, 49, 54, 64)	2 (27, 34)
	Clinical department	5 (23, 30, 54, 56, 57)	0
	Specialist nurse status, and nurse manager or clinical educator status	4 (34, 38, 49, 54)	0
AI technical perception and experience	AI perception, level of understanding, or knowledge	11 (26, 27, 30, 34, 38, 43, 48, 52, 53, 55, 63)	5 (27, 30, 34, 38, 43)
	AI training experience	5 (34, 38, 48, 50, 61)	1 (38)
	AI usage experience	8 (25, 27, 30, 38, 49, 53, 58, 63)	1 (30)
	Subjective perceived benefits	2 (53, 63)	0
Psychological and personal traits	AI anxiety	2 (43, 63)	5 (30, 34, 43, 58, 63)
	Digital leadership	1 (25)	2 (25, 27)
	Cognitive flexibility or emotion regulation	1 (59)	0

**Table 3:** Factors influencing artificial intelligence literacy.

Category of factors	Factors	Univariate analysis (*n*, references)	Multivariate analysis (*n*, references)
Demographic factors	Educational level	4 (33, 54, 60, 62)	0
	Years of work experience	1 (42)	0
	Age	2 (16, 60)	0
	Gender	2 (43, 58)	0
	Marital status	2 (60, 62)	0
Organizational and occupational characteristics	Leadership style	1 (42)	1 (42)
	Clinical department	3 (40, 54, 60)	0
	Hospital grade or type	2 (54, 60)	0
	Province	1 (54)	0
AI technical perception and experience	AI training experience	3 (40, 42, 54)	2 (40, 42)
	AI usage experience or frequency	4 (16, 40, 54, 58)	1 (42)
	Perceived subjective benefits and risks of AI	2 (16, 62)	0
	Information technology competence	1 (16)	0

#### 3.3.2 Organizational and occupational characteristics

Organizational and occupational characteristics influencing registered nurses’ artificial intelligence attitudes included hospital grade or type (*n* = 8), clinical department (*n* = 5), specialist nurse status, and nurse manager or clinical educator status (*n* = 4). Factors confirmed by multivariate regression analysis to have independent effects included hospital grade or type (*n* = 2) (see Table 2). Nurses in tertiary hospitals held more positive attitudes toward artificial intelligence than those in secondary or lower tier hospitals. Clinical department and manager or educator status showed no independent effect on nurses’ attitudes toward artificial intelligence. Factors associated with artificial intelligence literacy levels included clinical department (*n* = 3), hospital grade or type (*n* = 2), leadership style (*n* = 1), and province (*n* = 1). Regression analysis demonstrated that leadership style (*n* = 1) was an independent influencing factor, where transformational, authentic, and transactional leadership styles were significantly associated with nurses’ AI literacy levels, whereas servant leadership showed no significant association (see Table 3).

#### 3.3.3 AI technical perception and experience

Technical perception and experience regarding AI represent critical factors influencing registered nurses’ attitudes toward artificial intelligence. Findings from this review indicate that AI perception, level of understanding, or knowledge (*n* = 11), AI training experience (*n* = 5), AI usage experience (*n* = 8), and subjective perceived benefits of AI (*n* = 2) affected nurses’ AI attitudes. Multivariate analysis confirmed that AI perception, level of understanding, or knowledge (*n* = 5), AI training experience (*n* = 1), and AI usage experience (*n* = 1) served as independent influencing factors on registered nurses’ artificial intelligence attitudes (see Table 2). Meanwhile, AI training experience (*n* = 3), AI usage experience or frequency (*n* = 4), perceived subjective benefits and risks of AI (*n* = 2), and information technology competence (*n* = 1) affected registered nurses’ artificial intelligence literacy levels. Results of multivariate analysis demonstrated that AI training experience (*n* = 2) alongside usage experience or frequency (*n* = 1) served as independent factors influencing artificial intelligence literacy. Findings across two studies revealed that nurses who had participated in AI training and possessed practical usage experience achieved higher levels of artificial intelligence literacy (see Table 3).

#### 3.3.4 Psychological and personal traits

Psychological and personal traits affecting nurses’ attitudes toward artificial intelligence included artificial intelligence anxiety (*n* = 2), digital leadership (*n* = 1), and cognitive flexibility or emotion regulation (*n* = 1). Results of regression analyses revealed that artificial intelligence anxiety (*n* = 5) and digital leadership (*n* = 2) were independent factors influencing nurses’ artificial intelligence attitudes. Multiple studies consistently demonstrated that nurses’ artificial intelligence anxiety served as a significant negative predictor of attitudes, Higher levels of digital leadership and technology trust among nurses were associated with more positive attitudes toward artificial intelligence (see Table 2).

## 4 Discussion

Nurses’ attitudes toward artificial intelligence (AI) and their levels of AI literacy play a crucial role in determining whether new technologies can be successfully implemented in clinical practice. Currently, the vast majority of studies focus primarily on investigating nurses’ AI attitudes and literacy levels, or comprehensively describing clinical application scenarios of AI, lacking a systematic integration of evidence. This review conducted a descriptive analysis of the regression analysis results regarding the factors influencing registered nurses’ AI attitudes and literacy across studies. The findings indicate that multidimensional factors—including sociodemographic characteristics, organizational environment, technical experience, and psycho-ethical aspects—exert independent effects on nurses’ AI attitudes and literacy. However, certain factors demonstrate substantial contradictions and heterogeneity across different study findings.

Regarding sociodemographic characteristics, most studies reported that younger nurses and those with shorter work tenure exhibit more positive attitudes toward AI. This may be attributed to younger generations, as digital natives, being more sensitive to emerging digital technologies. Their frequent exposure to smart devices in daily life has fostered a higher baseline of technology acceptance. Nurses with lower tenure are in a stage of rapid learning and career advancement, making them more likely to view AI as a tool to enhance efficiency and reduce repetitive tasks. Similar findings have been reported in previous studies (23–25). Conversely, studies by Chen et al. (27) and Alrashedi et al. (26) indicated that older nurses and those with higher tenure possess a stronger willingness to use AI. Another multicenter survey supported this view, noting that nurses with longer years of service show a higher willingness to utilize AI in clinical practice (28). Older nurses possess richer clinical experience, which may allow them to perceive the value of AI technology in improving workflow efficiency and nursing care quality more intuitively, thereby demonstrating higher expectations. Nevertheless, this discrepancy may also be influenced by objective factors such as specific healthcare settings, imbalanced group sample sizes, and variations in measurement tools and data collection timing. We also observed that the cutoff values for grouping work tenure and age were inconsistent across studies, and sample distributions were skewed toward certain tenure ranges; thus, the interpretation of this factor warrants further verification. Regarding educational attainment, studies consistently showed that nurses with higher education levels hold more positive attitudes toward AI. Higher education plays a pivotal role in cultivating evidence-based thinking, information literacy, and critical analysis skills, enabling highly educated nurses to evaluate the direct benefits of complex technologies more objectively (29). Two studies demonstrated that male nurses exhibited significantly higher positive attitudes toward AI than female nurses (26, 30), a finding aligned with the study by Erkayıran et al. (23), potentially because males tend to be more active in exploring new technologies (31). Conversely, the study by Li et al. (32) reported more positive attitudes among female nurses. However, this contradiction likely stems largely from the inherently extreme gender imbalance within the nursing population and methodological limitations. In surveys targeting the nursing field, female samples often account for 80–90%. Such extreme gender proportions can lead to unstable regression coefficient estimates, thereby limiting the generalizability of these conclusions. The impact of professional titles or nursing career levels on attitudes also displayed a bidirectional nature. Studies by Li et al. (32) and Alrashedi et al. (26) indicated that nurses with lower professional titles held more positive attitudes toward AI. Junior nurses typically undertake a large amount of foundational clinical work and are more eager to reduce workload intensity through smart technology. In contrast, senior nurses may assume nursing management or teaching responsibilities, focusing more on the application of AI in quality control and decision support (33). This bidirectional positivity reflects differentiated demands regarding the functional positioning of AI across different career levels. Among organizational and occupational characteristics, hospital grade or category served as an independent influencing factor. Nurses in tertiary hospitals demonstrated more positive attitudes toward AI than those in secondary and lower-level hospitals (32, 34). This not only reflects disparities in medical resource allocation but also underscores the profound impact of regional digitalization levels. Tertiary hospitals generally possess stronger financial support and more comprehensive medical information technology infrastructure, providing nurses with greater opportunities for exposure to cutting-edge technologies. In contrast, primary care hospitals remain on the periphery of technological reform due to resource constraints and related issues. This study also found a high concentration in the geographical distribution of the included primary studies. Due to objective differences in healthcare systems and the progress of digitalization across countries, in environments with severe human resource shortages, nurses’ urgent need for automated workload-reduction tools may overshadow their resistance to technology. Conversely, in regions emphasizing humanistic nursing care and stringent ethical review, nurses’ bias against algorithms and distrust regarding privacy and security may be more pronounced. A study by Baraka et al. (35) also pointed out that external conditions, including resource support, significantly influence nurses’ readiness to adopt AI. Cognition and usage experience regarding AI technology are core factors influencing nurses’ attitudes toward AI; nurses who have a deeper understanding, higher level of cognition, and prior usage experience with AI hold more positive attitudes. A deeper comprehension of AI algorithmic principles and application advantages can mitigate distrust stemming from insufficient algorithmic explainability and data privacy risks (36). The positive effect of prior usage experience on attitudes suggests that technophobia often originates from unfamiliarity, and increasing exposure through simulation training is an effective pathway to eliminate anxiety (37). However, constrained by the homogeneity defect of measurement tools, the estimated effect size of technical experience fluctuated across studies. Most included primary studies utilized generic AI attitude or literacy scales, treating AI as a single, homogenized concept without distinguishing whether nurses were exposed to simple administrative support software or highly complex decision support systems. This may explain the variations in attitude effect sizes observed across different studies. Nurses with AI training experience exhibited higher positive attitudes toward AI (30, 38). Studies by Qaladi et al. (39) and Rony et al. (9) emphasized that comprehensive training plays a vital role in the successful integration of AI technology with clinical practice. An intervention study comparing nurses’ AI attitudes before and after professional AI training revealed that the proportion of nurses holding positive attitudes increased from 16.3 to 82.8% post-training.

Furthermore, AI training experience and prior usage experience serve as essential factors influencing registered nurses’ AI literacy. The included literature consistently indicates that nurses who have participated in training or possess prior usage experience demonstrate higher levels of AI literacy. Targeted educational training can enhance nurses’ information-retrieval capabilities, leading to more prominent competence in technology utilization and evaluation. Relevant training not only strengthens nurses’ theoretical understanding of cutting-edge technologies but also effectively improves their clinical application capabilities through simulation and hands-on practice (40, 41). However, synthesizing existing studies reveals that the current proportion of nurses who have received AI-related training remains generally low (16, 40, 42, 43). Meanwhile, nurses’ experience in using smart devices builds a certain foundation for adopting new technologies (23). Sommer et al. (15) also pointed out that past digital experience forms the benchmark for technological transition, with nurses who are more familiar with intelligent tools exhibiting higher AI literacy. Nurses with higher literacy levels are better equipped to keenly appreciate the value of AI technology in assisting clinical workflows, thereby holding a more positive stance toward the technology. A positive AI attitude can, in turn, drive nurses to proactively acquire relevant knowledge and skills, which subsequently translates into higher AI literacy (33). The study by Tong et al. (42) also noted that managers’ leadership styles impact registered nurses’ AI literacy. Among these, transformational, transactional, and authentic leadership styles are significantly positively correlated with nurses’ literacy levels. Transformational leaders advocate innovation and proactively take on risks associated with implementing new technologies, creating a psychologically safe environment for nurses (44). Transactional leaders provide external motivation and resource exchange conditions for nurses to learn new technologies by establishing clear reward and punishment systems (45). Authentic leaders fulfill nurses’ basic needs and respect their dedication during the process of learning new technologies, thereby stimulating their intrinsic drive to improve literacy (46). Hospitals should develop comprehensive leadership training programs to help nursing managers flexibly employ diverse leadership strategies (42). Nursing managers should integrate AI literacy education into the continuing education system for nurses, specifically increasing hands-on components in real-world clinical scenarios to further boost nurses’ AI attitudes and self-efficacy, thereby facilitating the successful implementation of technology (33).

Regression analysis results across multiple studies consistently indicate that AI anxiety and ethical dilemmas exert a significant negative impact on nurses’ AI attitudes and literacy. While the introduction of AI technology alleviates workloads, frequent system alarms and complex operating procedures are prone to triggering alarm fatigue and technostress, thereby escalating nurses’ cognitive load and psychological exhaustion. Studies have noted that the ethical challenges nurses currently face have expanded from mere data privacy to the level of professional identity; nurses generally harbor concerns that excessive AI involvement will replace their jobs, or that their inability to keep pace with technological development will lead to social marginalization (35, 47). When inherent biases exist in AI algorithms, nurses, as the ultimate executioners of clinical decisions, confront decision-making conflicts between algorithms and clinical experience. However, legal liability and ethical accountability mechanisms for algorithm-induced biases remain ill-defined at present. This contradiction between perceived utility and potential risks may induce resistance among nurses toward introducing AI technologies into clinical practice. When exploring the perspectives of nursing professionals, Rony et al. (9) also thoroughly discussed the importance of ethical considerations during technology implementation. Higher levels of perceived digital leadership and technology trust among nurses correlate with more positive AI attitudes. The outstanding digital leadership demonstrated by nursing managers can foster a psychologically safe environment for the team; by offering a clear vision for digital transformation and ample resource support, it effectively alleviates nurses’ technostress, thereby enhancing nurses’ acceptance of new technologies at the organizational level (25, 27).

### 4.1 Strengths and limitations

This study strictly adheres to the PRISMA reporting guidelines. The protocol was prospectively registered on the PROSPERO platform, and the standardized JBI tool was utilized to evaluate the quality of the included studies, ensuring the transparency, rigor, and reliability of the research process and results. A systematic search across 10 major English and Chinese databases was conducted, enhancing the comprehensiveness of the evidence. Independent factors influencing registered nurses’ AI attitudes and literacy were extracted and categorised by theme, providing an important empirical basis for future targeted educational training and intervention strategies. However, this study also has several limitations. All included studies were cross-sectional surveys, which can only reveal associations between variables rather than establish causal relationships between influencing factors and nurses’ attitudes or literacy. Given the substantial heterogeneity among the included studies in terms of outcomes, measurement tools, and participant characteristics, a narrative synthesis approach was adopted instead of a meta-analysis. Furthermore, this review was restricted to literature published in English and Chinese, potentially missing relevant studies in other languages. Additionally, the included literature was geographically concentrated primarily in China and Turkey, which may introduce geographical concentration and language bias; caution is therefore warranted when generalizing these findings to countries with different cultures or healthcare systems. Finally, constrained by the methodological design of the included primary studies—most of which investigated nurses’ attitudes and literacy using a generalized concept of artificial intelligence—subgroup analyses or nuanced discussions tailored to specific types of AI technologies could not be performed.

## 5 Conclusion

This study is the first to systematically synthesize the independent factors influencing registered nurses’ artificial intelligence attitudes and literacy. The findings reveal that among sociodemographic characteristics, only educational attainment demonstrated a consistent impact on nurses’ AI attitudes across studies. The remaining factors exhibited substantial discrepancies across study results, and their exact underlying mechanisms warrant further verification in future research. Notably, after controlling for variables such as usage experience and training history, sociodemographic factors—including educational attainment, work tenure, and age—failed to show independent effects on nurses’ AI literacy in the regression analyses across studies. This finding suggests that nurses’ mastery of AI technology may depend more on post-hoc interventions and cultivation rather than inherent baseline characteristics. Therefore, healthcare institutions should avoid demographic stereotypes in clinical practice and commit to optimizing human-computer interaction interfaces. Policy makers must establish robust data privacy protection regulations and ethical guidance frameworks, clearly defining the allocation of legal liabilities for AI technologies. Specialized training in algorithm bias recognition and ethics education should be strengthened to alleviate nurses’ concerns regarding algorithmic bias and job displacement, thereby fostering trust in technology while safeguarding humanistic care and ensuring nurses can refocus more energy on patient care with technological support. Defining nurses’ roles and positions in the digitization process is essential to promote the healthy development of the nursing profession. Given the currently low proportion of nurses who have received AI training, managers should vigorously implement AI-related training to facilitate the effective integration of artificial intelligence in nursing. Building targeted AI continuing education systems with simulation training and hands-on coursework can bridge knowledge gaps and enhance nurses’ critical evaluation capabilities regarding technological logic. Furthermore, this study indicates that existing nursing research mostly treats AI as a homogeneous construct, which may obscure nurses’ genuine attitudes toward and demands for specific AI tools. Future research should focus on investigating specific AI application scenarios to more precisely capture nurses’ attitudes and literacy requirements regarding different technological tools. Additionally, owing to the marked heterogeneity among current study findings and the absence of standardized measurement tools, future studies should develop standardized assessment tools and conduct multicenter, large-sample longitudinal research to deeply explore the long-term effects of educational and clinical interventions, as well as shifts in nurses’ attitude and literacy levels across diverse cultural contexts, ultimately achieving the safe, efficient, and sustainable integration of AI technology in the nursing field.

## Data Availability

The original contributions presented in the study are included in the article/Supplementary material, further inquiries can be directed to the corresponding author.
