# Supplementary material for: Factors influencing registered nurses’ attitudes toward and literacy in artificial intelligence: a cross-sectional systematic review
Source: Front Public Health. 2026 Oct 23;14:1997701. doi: 10.3389/fpubh.2026.1997701 (PMC13740691; doi:10.3389/fpubh.2026.1997701)
Supplement: Supplementary file 1 [file Table_1.docx]

Supplementary Material

**Supplementary Tables A** **Retrieval strategy**

| Database | Search Strategy | Filters |
| --- | --- | --- |
| PubMed/Medline | ("Nurses"[Mesh] OR "registered nurse*"[Title/Abstract] OR "nurs*"[Title/Abstract] OR "nursing staff"[Title/Abstract] OR "clinical nurse"[Title/Abstract])AND("knowledge"[Title/Abstract] OR "awareness"[Title/Abstract] OR "understanding"[Title/Abstract] OR "literacy"[Title/Abstract] OR "attitude*"[Title/Abstract] OR "perception*"[Title/Abstract] OR "view"[Title/Abstract] OR "opinion"[Title/Abstract] OR "acceptance"[Title/Abstract] OR "readiness"[Title/Abstract] OR "willingness"[Title/Abstract] OR "adoption"[Title/Abstract])AND("Artificial Intelligence"[Mesh] OR "Machine Learning"[Title/Abstract] OR "Deep Learning"[Title/Abstract] OR "Neural Network*"[Title/Abstract] OR "Natural Language Processing"[Title/Abstract] OR "ChatGPT"[Title/Abstract] OR "large language model*"[Title/Abstract] OR "AI"[Title/Abstract]) | English |
| Web of Science | TS=( AI terms )  AND TS=( nurse terms )  AND TS=( acceptance terms ) | English |
| Embase/Cochrane | ("nurses" OR "registered nurse*" OR "nurs*" OR "nursing staff" OR "clinical nurse"):ti,ab,kw  AND("knowledge" OR "awareness" OR "understanding" OR "literacy" OR "attitude*" OR "perception*" OR "view" OR "opinion" OR "acceptance" OR "readiness" OR "willingness" OR "adoption"):ti,ab,kw  AND("artificial intelligence" OR "machine learning" OR "deep learning" OR "neural network*" OR "natural language processing" OR "chatgpt" OR "large language model*" OR "ai"):ti,ab,kw | English |
| IEEE | Full-text search: AI terms AND nurse terms AND acceptance terms | English |
| CINAHL | MH “Artificial Intelligence+” OR TI/AB AI terms  AND Nursing terms: MH “Nurses+” OR TI/AB nurse terms  AND Acceptance terms: TI/AB acceptance terms | English |
| CNKI, Wanfang, VIP | ("Nurses" OR "Nursing Staff"OR "Clinical Nurses" OR "Registered Nurses") [Subject]  AND("Knowledge" OR "Attitudes" OR "Cognition" OR "Views" OR "Perceptions" OR "Literacy" OR "Acceptance") [Subject]  AND("Artificial Intelligence" OR "Machine Learning" OR "Deep Learning" OR "Natural Language Processing" OR "Neural Networks" OR "ChatGPT" OR "Large Language Models") [Subject] |  |

**Key term groups**

**nurse terms:** "Nurses" OR "registered nurse*" OR "nurs*" OR "nursing staff" OR "clinical nurse"

**Attitude and Literacy terms:** "Knowledge" OR "Awareness" OR "Understanding" OR "Literacy" OR "attitude*" OR "perception*" OR "View" OR "Opinion" OR "Acceptance" OR "Readiness" OR "Willingness" OR "adoption"

**AI terms:**"Artificial Intelligence" OR "Machine Learning" OR "Deep Learning" OR "Neural Network*" OR "Natural Language Processing" OR "ChatGPT" OR "large language model*" OR "AI"

**Supplementary Tables B Quality Assessment (JBI Critical Appraisal – Analytical Cross-Sectional Studies)**

| Study | 1. Inclusion criteria defined? | 2. Subjects & setting described? | 3. Exposure measured validly & reliably? | 4. Standard criteria for outcome measurement? | 5. Confounders identified? | 6. Strategies to deal with confounding? | 7. Outcomes measured validly & reliably? | 8. Appropriate statistical analysis? | Total Score | Max Score | QA Rating |
| --- | --- | --- | --- | --- | --- | --- | --- | --- | --- | --- | --- |
| Deng et al,2025 | Yes | Yes | Yes | Yes | Yes | Yes | Yes | Yes | 8 | 8 | High |
| Kong et al,2025 | Yes | Yes | Yes | Yes | Yes | Yes | Yes | Yes | 8 | 8 | High |
| Liu et al,2026 | Yes | Yes | Yes | Yes | Yes | Yes | Yes | Yes | 8 | 8 | High |
| Taşçı et al,2026 | Yes | Yes | Yes | Yes | No | No | Yes | Yes | 6 | 8 | Moderate |
| Kahraman et al,2025 | Yes | Yes | Yes | Yes | No | No | Yes | Yes | 6 | 8 | Moderate |
| Lin et al,2025 | Yes | Yes | Yes | Yes | Yes | Yes | Yes | Yes | 8 | 8 | High |
| Tarsuslu et al,2025 | Yes | Yes | Yes | Yes | Yes | Yes | Yes | Yes | 8 | 8 | High |
| Atalla et al,2025 | Yes | Yes | Yes | Yes | Yes | Yes | Yes | Yes | 8 | 8 | High |
| Erkayıran et al,2025 | Yes | Yes | Yes | Yes | No | No | Yes | Yes | 6 | 8 | Moderate |
| Alruwaili et al,2024 | Yes | Yes | Yes | Yes | No | No | Yes | Yes | 6 | 8 | Moderate |
| Li et al,2026 | Yes | Yes | Yes | Yes | Yes | Yes | Yes | Yes | 8 | 8 | High |
| Sayed et al,2026 | Yes | Yes | Yes | Yes | No | No | Yes | Yes | 6 | 8 | Moderate |
| Xu et al,2026 | Yes | Yes | Yes | Yes | Yes | Yes | Yes | Yes | 8 | 8 | High |
| Alrashedi et al,2026 | Yes | Yes | Yes | Yes | Yes | Yes | Yes | Yes | 8 | 8 | High |
| Tuncer et al,2024 | Yes | Yes | Yes | Yes | No | No | Yes | Yes | 6 | 8 | Moderate |
| Zeng et al,2025 | Yes | Yes | Yes | Yes | Yes | Yes | Yes | Yes | 8 | 8 | High |
| Namdar et al,2025 | Yes | Yes | Yes | Yes | No | No | Yes | Yes | 6 | 8 | Moderate |
| Cin et al,2026 | Yes | Yes | Yes | Yes | No | No | Yes | Yes | 6 | 8 | Moderate |
| Gürdap et al,2026 | Yes | Yes | Yes | Yes | Yes | Yes | Yes | Yes | 8 | 8 | High |
| Liu et al,2025 | Yes | Yes | Yes | Yes | Yes | Yes | Yes | Yes | 8 | 8 | High |
| Hacıalioğlu et al,2025 | Yes | Yes | Yes | Yes | Yes | Yes | Yes | Yes | 8 | 8 | High |
| Özçevik Subaşi et al,2025 | Yes | Yes | Yes | Yes | Yes | No | Yes | Yes | 7 | 8 | High |
| Alnazly et al,2026 | Yes | Yes | Yes | Yes | Yes | Yes | Yes | Yes | 8 | 8 | High |
| Çoban et al,2026 | Yes | Yes | Yes | Yes | No | No | Yes | Yes | 6 | 8 | Moderate |
| Chen et al,2025 | Yes | Yes | Yes | Yes | Yes | Yes | Yes | Yes | 8 | 8 | High |
| Atalla et al;2024 | Yes | Yes | Yes | Yes | Yes | Yes | Yes | Yes | 8 | 8 | High |
| Uludaşdemir et al;2026 | Yes | Yes | Yes | Yes | Yes | Yes | Yes | Yes | 8 | 8 | High |
| Nirgiz et al;2026 | Yes | Yes | Yes | Yes | Yes | Yes | Yes | Yes | 8 | 8 | High |
| Tong et al;2026 | Yes | Yes | Yes | Yes | Yes | Yes | Yes | Yes | 8 | 8 | High |
| Yu et al;2025 | Yes | Yes | Yes | Yes | Yes | Yes | Yes | Yes | 8 | 8 | High |

**Quality Assessment Checklist：**

1. Were the criteria for inclusion in the sample clearly defined?
2. Were the study subjects and the setting described in detail?
3. Was the exposure measured in a valid and reliable way?
4. Were objective, standard criteria used for measurement of the condition?
5. Were confounding factors identified?
6. Were strategies to deal with confounding factors stated?
7. Were the outcomes measured in a valid and reliable way?
8. Was appropriate statistical analysis used?
